# Two-step closure of the Miocene Indian Ocean Gateway to the Mediterranean

**DOI:** 10.1038/s41598-019-45308-7

**Published:** 2019-06-20

**Authors:** Or M. Bialik, Martin Frank, Christian Betzler, Ray Zammit, Nicolas D. Waldmann

**Affiliations:** 1 Dr. Moses Strauss Department of Marine Geosciences, The Leon H. Charney School of Marine Sciences, University of Haifa, Carmel, 31905 Israel; 2 GEOMAR Helmholtz Centre for Ocean Research Kiel, Kiel, Germany; 3 Institute of Geology, CEN, University of Hamburg, Bundesstrasse 55, Hamburg, 20146 Germany; 4 The School of Earth and Ocean Sciences, Cardiff University, Main Building, Parc Place, Cardiff, CF10 3AT UK

**Keywords:** Palaeoceanography, Palaeoclimate

## Abstract

The Tethys Ocean was compartmentalized into the Mediterranean Sea and Indian Ocean during the early Miocene, yet the exact nature and timing of this disconnection are not well understood. Here we present two new neodymium isotope records from isolated carbonate platforms on both sides of the closing seaway, Malta (outcrop sampling) and the Maldives (IODP Site U1468), to constrain the evolution of past water mass exchange between the present day Mediterranean Sea and Indian Ocean via the Mesopotamian Seaway. Combining these data with box modeling results indicates that water mass exchange was reduced by ~90% in a first step at ca. 20 Ma. The terminal closure of the seaway then coincided with the sea level drop caused by the onset of permanent glaciation of Antarctica at ca. 13.8 Ma. The termination of meridional water mass exchange through the Tethyan Seaway resulted in a global reorganization of currents, paved the way to the development of upwelling in the Arabian Sea and possibly led to a strengthening of South Asian Monsoon.

## Introduction

The Tethyan Ocean dominated global ocean circulation during most of the Mesozoic and Cenozoic. This low latitude ocean basin allowed water mass exchange between the Atlantic, Indian and Pacific oceans that resulted in a global climate system markedly different than that of today. This low latitude circumglobal connection across the Tethys Ocean had a significant effect on both global heat transfer and nutrient availability in the tropics and lower sub-tropics^1^. Saline deep and intermediate waters generated by intense evaporation at low latitudes in the Tethys realm^2–4^ were the principal engine for the global halothermal overturning circulation. This mode of overturning circulation would have been the main driver of global oceanic heat transfer in the absence of a thermohaline circulation driven by large permanent ice sheets in the polar regions.

Northward movement of the African, Australian and Indian plates, which started during the Late Cretaceous (ca. 80 Ma) and persisted until the Oligocene, resulted in a continuous narrowing of the vast Tethys basin and eventually led to the formation of the enclosed, marginal Mediterranean and Black Seas. The initial disconnection of the Mediterranean realm from the world oceans occurred at the eastern end of the present Mediterranean Sea and resulted in the termination of the low latitude circumglobal ocean circulation. This connection allowed transport of Indian Ocean surface waters to the Atlantic Ocean and at the same time subsurface waters were advected to the Indian Ocean^5,6^. The newly formed enclosed Mediterranean Sea subsequently experienced intervals of anoxic and suboxic conditions of its deep waters resulting in sapropel deposition. Ultimately, restriction of exchange with the Atlantic Ocean paved the way for a complete closure and evaporation of the Mediterranean Sea during the Late Miocene Messinian Salinity Crisis^7,8^. Contemporaneous to the closing of the Indian Ocean – Mediterranean connection the modern circulation of the Arabian Sea was established, which boosted the South Asian Monsoon (SAM) in the Indian Ocean. Moreover, the termination of the low latitude seaway connecting the Atlantic and Indian oceans had major consequences for both the Atlantic Meridional Overturning Circulation (AMOC) as well as the Antarctic Circumpolar Current (ACC) and therefore played an important role in major global oceanic reorganisation^5^. However, despite the significance of this global event, its exact timing and the involved sequence of tectonic and oceanographic changes are still debated.

Paleontological data, primarily based on slowly evolving macrofaunal distribution and tectonic considerations^9–12^ have allowed to broadly constrain the timing of the closure of the Mesopotamian Seaway (which represents the sub-basin of the Tethyan Seaway north of the Arabian Plate) and the termination of a continuous Tethyan Seaway connectivity between and Indian and Atlantic Oceans to a time between the Late Oligocene and Middle Miocene (ca. 23 - 14 Ma). A second connection across the Red Sea may still have existed until at least the Aquitanian (20 Ma^13,14^) but the feasibility and evolution of significant exchange of water masses between the Mediterranean Sea and Indo-Pacific oceans is still debated. With this disconnection, surface waters were no longer exchanged between the Indian and the Atlantic Oceans at low latitudes, and Mediterranean Intermediate Waters were from thereon only advected to the Atlantic Ocean.

Here we present a paleoceanographic approach which allows the reconstruction of water mass exchange between the Indian Ocean and the Mediterranean across the Tethyan Seaway based on radiogenic Nd isotopes. This is enabled by the intermediate residence time of Nd in seawater (between 200 and 1500 years) and the isotopic labelling of waters via continental inputs at the ocean margins, resulting in distinct Nd isotope signatures of water masses^15^. Similar studies have been used to infer that the final closure of deep water mass exchange across the Central American Seaway was established ca. 5 Ma^16^ and that subsequent Nd isotope shifts in the Atlantic Ocean were a consequence of linked changes in Atlantic circulation and continental weathering inputs^17–19^. In the case of the Mediterranean, previously published Nd isotope records from northern Italy have been inferred to reflect a connection between the Indian Ocean and the Mediterranean Sea until the Langhian-Serravallian (14 to 13 Ma)^20,21^. These studies showed that ɛNd signatures of Mediterranean water masses were more radiogenic than their present-day values during periods of connection to the Indian Ocean, which has been attributed to volcanic contributions from either northern Arabia or the Central Mediterranean. Some of those volcanic deposits are interbedded in the previously studied section in Northern Italy and may have contributed to the observed signal. This, however, is in contradiction to the timing established by other evidence, such as for the “Gomphotherium landbridge” and by other markers from the fossil record such as terrestrial land animals, corals (now in inland positions) and gastropods^9,22,23^.

The goal of this study was to refine previous estimations based on two new Nd isotope datasets from isolated carbonate platforms far from adjacent volcanic or immediate terrestrial contributions in both the Mediterranean Sea (Malta) and in the western Indian Ocean (Maldives). Combining these results with a simple box modelling approach to calculate seawater Nd isotope values as a function of through-flow strength across the East Tethyan/Mesopotamian gateway allows to (i) globally correlate stratigraphic constraints on the changes in connectivity and (ii) to estimate the magnitude of change in the volume flow of water across the seaway. Our results suggest a major drop of Indian Ocean water mass contributions to the Mediterranean by one order of magnitude during the early Burdigalian (ca. 19.7 Ma), likely followed by a period of restricted exchange persisting until the late Langhian (ca. 13.8 Ma). The connection was finally terminated following the Langhian-Serravallian transition, which coincided with the onset of permanent glaciation of Antarctica^24^.

## Geological setting

**Figure 1:**
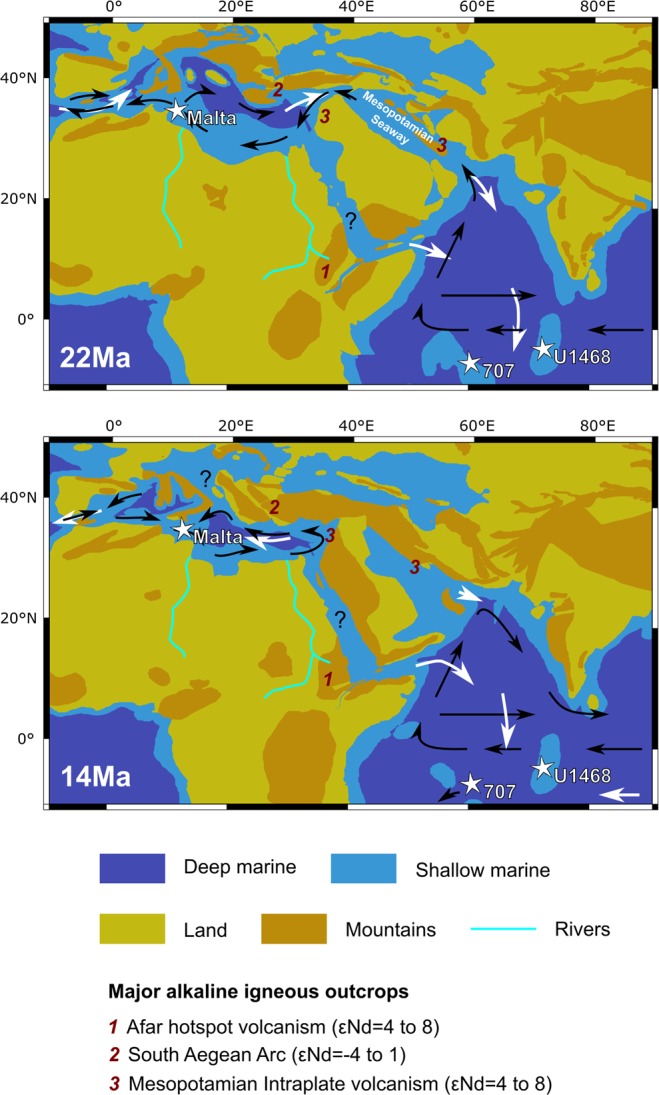
Early to Middle Miocene paleogeography of the Mediterranean Sea and western Indian Ocean^13^: Arrows indicate surface circulation patterns^6,12,101^, with black arrow indicate surface waters and white intermediate to deep waters. Red numbers indicate potential source areas of runoff with positive εNd signatures. Major drainage systems of the northern African continent are marked. The speculated area of the “Gomphotherium landbridge” overlaps with the Mesopotamian Seaway.

For this study, two isolated carbonate platform sites at both sides of the Tethyan Seaway were chosen (Fig. 1): The Maldives in the Indian Ocean and Malta in the Mediterranean.

## Maldives

The Maldives are a paired chain of atolls in the equatorial western Indian Ocean, some 100 km wide (east-west) and 1000 km long (north-south). These atolls are the surface manifestation of a large isolated carbonate platform that formed on a Paleocene magmatic ridge^25^. Continuous calcareous sediments accumulated on this ridge resulting in a near complete Cenozoic sedimentary succession without any significant terrigenous influence^26^. The succession comprises two principal depositional phases: a pre-monsoonal high deposition rate platform from the Oligocene until 13 Ma, followed by a monsoonal, drift dominated, depositional phase from 13 Ma to present^27,28^.

## Malta

The Maltese archipelago is the exposed part of a carbonate platform that developed from the Paleocene until the Messinian salinity crisis^29^. The Oligocene-Miocene succession in the archipelago comprises a mostly recrystallised neritic platform (Lower Coralline Limestone Fm.), which subsequently drowned by rising sea level and development of a carbonate factory (Globigerina Limestone Fm.) during the Late Oligocene to the Middle Miocene. This calcareous dominated periplatform sequence was occasionally punctuated by phosphatic events^30^, which were deposited until the Langhian-Serravallian boundary (13.82 Ma). An increase in detrital input followed the Langhian-Serravallian transition, expressed in a lithological transition from limestone to marls (Blue Clay Fm.), which were subsequently capped by a late Serravallian to Tortonian unconformity (Green Sand Fm.). Following this unconformity, there was a second phase of neritic carbonate deposition (Upper Coralline Limestone Fm.).

## Methods

### Sampling

IODP Expedition 359 conducted scientific coring at the periphery of a drowned carbonate platform in the Maldives inner sea in 2015^27,31^. Two Sites along the northern transect, U1466 and U1468, penetrated a substantial part of the Early-Middle Miocene pre-drowning sequence. Site U1468 (4°55.98′N/73°4.28′E) reached the oldest sediments including the Oligocene-Miocene boundary above its base, representing the most extensive and well dated Early to Middle Miocene sedimentary record to date in the Northwestern Indian Ocean^28,32–36^. The paleo water depth during deposition of these sediments was on the order of several hundreds of meters at most. Age model and subdivision of the sequences of Site U1468 are adopted from prior studies^37^. A total of 28 samples of calcareous material from the peri-platform sequence of Site U1468 were used in this study and serve as our Indian Ocean reference record.

Samples from Malta were collected from the il-Blata section (35°54.01′N/14°19.88′E)^38^, from the Gnejna Bay section (35°55.57′N/14°20.63′E), adjacent to Ras il-Pellegrin^39^, and from the Fomm ir-Rih Bay section (35°54.36′N/14°20.46′E). The latter has also been the subject of a previous Nd isotope study^40^. The paleobathymetry during deposition of these sediments also was on the order of several hundreds of meters^41^. The age model is based on established biostratigraphic and ^87^Sr/^86^Sr-based ages for the Ras il-Pellegrin, Fomm ir-Rih and il-Blata sections^38,39,42^. A total of 12 samples of calcareous material were used for this study as our Mediterranean reference record.

### Nd isotope analysis

Samples were powdered, rinsed and digested in a 0.05 M hydroxylamine hydrochloride/15% acetic acid solution, buffered with NaOH to a pH of 4. The supernatant was separated and passed through cation exchange columns with 0.8 ml AG50W-X12 resin followed by 2 ml Ln-Spec resin to separate Nd from other cations and Rare Earth Elements, respectively^43,44^. Neodymium isotope ratios were measured on a Neptune Multiple Collector Inductively Coupled Plasma Mass Spectrometer (MC-ICPMS) at GEOMAR Kiel, Germany. Measured ^143^Nd/^144^Nd results were mass-bias corrected to a ^146^Nd/^144^Nd ratio of 0.7219 and were normalized to the accepted ^143^Nd/^144^Nd value of 0.512115 for the JNdi-1 standard^45^. Nd isotope ratios are reported as *ε*Nd_(*t*)_ values, which represent deviations from the Chondritic Uniform Reservoir (CHUR) and are calculated as εNd_(*t*)_ = [(^143^Nd/^144^Nd)sample(*t*)/(^143^Nd/^144^Nd)CHUR(*t*) − 1] * 10^4^ using a (^143^Nd/^144^Nd)CHUR(0) value of 0.512638. Details of the chemical preparation and analysis are provided in the supplement and the data are provided in Table S1.

### Modeling

To obtain a coarse estimation of the ɛNd values resulting from an open and closed connection between the Indian Ocean and the Mediterranean, a simple one box model was constructed, with the aim to approximate certain aspect of the system. The model was then tuned with possible variations and contributions from different input sources. A full description of the model and its parameterizations is given in the supplement.

## Results and Discussion

### The Maldives and Maltese records

**Figure 2:**
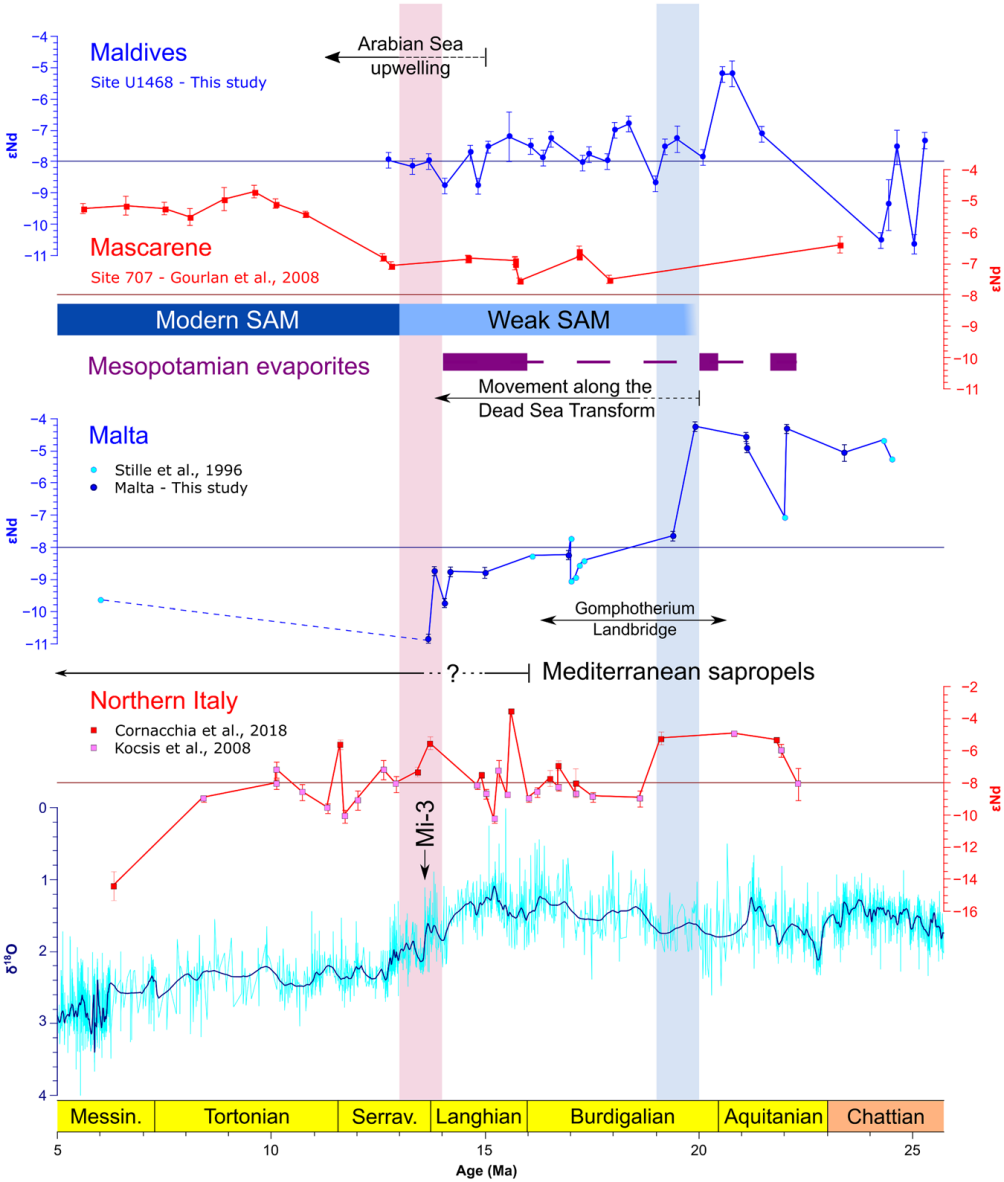
ɛNd records of the western Indian Ocean from IODP Site U1468 (this study; Indian Ocean upper water column), ODP Site 707^101^ (Indian Ocean intermediate waters) and of the Central Mediterranean from Malta (this study & Stille *et al*., 1996; Mediterranean upper water column) and northern Italy^20,21^ (Paratethys marginal upper water column). The composite Pacific benthic foraminiferal δ^18^O record^107^ is provided as a measure of sea level change. Line bars note: onset of Western Arabian Sea upwelling^86,87^; initiation and intensification of SAM^27,28^; initiation of emplacement of evaporites in Iraq and S. Iran and Syria^9,22,90^; initiation of movement along the Dead Sea Transform^81^; Gomphotherium landbridge^22,23^ and emplacement of Mediterranean sapropels^84,85^.

The Maldives Nd isotope record can be subdivided into three intervals (Fig. 2): the Chattian to Aquitanian (26–21 Ma), characterized by variable ɛNd signatures ranging from −7.3 ± 0.3 to −10.6 ± 0.3; the Late Aquitanian (20.5–20.8 Ma), marked by more radiogenic ɛNd signatures <−5.2 ± 0.4; and the Burdigalian to Serravallian (19–13 Ma), represented by a stable interval with a mean ɛNd value of −7.8 ± 0.6. The Middle Miocene values are essentially indistinguishable from modern Indian Ocean seawater values (−8.0 ± 1.1^46,47^) and from ferromanganese crust records^18^. The post-Aquitanian values are only slightly less radiogenic than those of intermediate water records from the Indian Ocean^37,48^.

The records from Malta are combined with results from previous studies^40^ and show persistent, highly radiogenic ɛNd values (mean of −4.6 ± 0.4) until ca. 20 Ma only interrupted by a possible short-term drop to a value of −7 at ca. 22 Ma (Fig. 2). Between 20 and 19 Ma, ɛNd signatures dropped and remained roughly constant at values of −8.8 ± 0.5 until 13.8 Ma when the signatures further dropped to values of −10.8 ± 0.1. The available data suggests that these remained steady until the late Miocene. These Miocene values differ from modern East Mediterranean seawater ɛNd signatures (−6 to −7^49^), while the post 20 Ma values exhibit some similarity to the modern Western Mediterranean ɛNd signatures (−8 to −10^49^). However, the Early Miocene values are much more radiogenic than expected from a simple mixture between Atlantic waters (ɛNd = −10.4 ± 0.8^49,50^) and Indian Ocean waters, either based on modern values or the ones observed at the Maldives (see modeling results in supplement). Moreover, the Middle to Late Miocene values do not match the expected pure Atlantic Ocean values, but it is possible that Atlantic water masses were more radiogenic at the time^40^. The implication of these observations is that the Mediterranean/Tethyan water mass did not obtain its ɛNd signatures by simple mixing of Indian and Atlantic Ocean waters but was rather modified by exchange with the coastal sediments derived from erosion of locally exposed rocks along their flow path^15^.

### Neodymium isotope labeling of mediterranean water masses

The modern Eastern Mediterranean sea ɛNd signature is much more radiogenic then its parent oceanic water source due to interactions at its margins^51^, yet is less radiogenic than the late Oligocene / Early Miocene ɛNd values recorded in Malta and northern Italy^20,21^ indicating differences in the contributing sources. The modern values result from weathering contributions of the Ethiopian highlands (via the Blue Nile) and the Aegean islands^51^. The alkaline volcanism that produced these source rocks is characterised by highly radiogenic ɛNd values between −5 and +5 ^52–54^. The Afar hotspot volcanism and the associated plume may have been established prior to the Eocene^55,56^, but it is unclear if any drainage system supplying weathering products from these volcanic rocks to the Mediterranean existed before the late Miocene^57^. The Aegean and western Anatolian volcanism had already been initiated during the Miocene^58,59^, yet it was still nascent at that time (with continued development until the present). If its Nd contributions to seawater had been significant at the time, we argue that the corresponding signal would have also been observable in the Middle and Late Miocene sections, which is not the case.

Another sequence of basaltic rocks exposed during the Late Oligocene and Early Miocene extends from Syria to southern Iran, bordering the inferred southern flanks of the Mesopotamian Seaway^60–63^. These rocks have ɛNd values of +1 to +5.6 and would probably have been more susceptible to erosion and weathering during the period of time the gateway was open, given that regional climate was more humid than today^64–66^. Furthermore, the local drainage system most probably provided waters to the Mesopotamian Seaway and the Mediterranean Sea. It was later diverted towards the Persian Gulf and the Arabian Sea as a consequence of the uplift of the Zagros Mountains during the Oligo-Miocene^67^. Highly radiogenic ɛNd signatures observed both at the Maldives during the late Aquitanian (ca. 21–22 Ma), may reflect a drainage pattern from the southern margins of the Mesopotamian Seaway re-routed into the Indian Ocean.

Another possible set of contributors are the Western and Central Mediterranean provinces. These are rich in Cenozoic volcanics, most of which are either younger than 10 Ma or older than 30 Ma^68–71^. The younger provinces would not have had an influence on the period of time considered in this study, but the effect of erosion and exchange with the older provinces on the results presented here cannot be excluded. This volcanic activity includes the evolution of the extension of the Tyrrhenian and Algéro-Provençal basins during the Miocene^72,73^ with its extensive seafloor spreading and its related hydrothermal activity. Deep water hydrothermal interactions at the flanks of a spreading centers do not generally contribute to the ɛNd signatures of near surface waters, with Nd being essentially quantitatively sequestered at the ridge flanks^74^. Thus, any contributions of radiogenic Nd isotope signatures from volcanics would have originated from the exposures in Mallorca and Sardinia^75,76^, which are, however, significantly less radiogenic (ɛNd values mainly between −2 and −9) then the northern Arabian sources.

**Figure 3:**
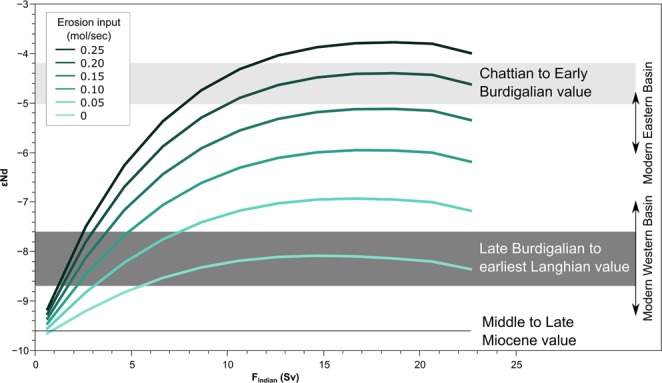
Modeled ɛNd values in the Mediterranean Sea for different volume inflow values from the Indian Ocean and erosional contributions from alkaline volcanic rocks along the Mesopotamian gateway.

Labelling of waters flowing in from the Indian Ocean with highly radiogenic Nd isotope signatures can explain the observed more positive ɛNd signatures found in the Maltese and northern Italian records during the Late Oligocene and Early Miocene. Based on inflow parameters obtained from modelling^6^ and the Nd isotope compositions in each oceanic basin (Table S1), we calculate the ɛNd signatures of the Mediterranean waters using a simple box model including the effect of Nd addition from, or exchange with the volcanic source rocks (Fig. 3). Using the prefered parameters, the erosional input rate required to achieve these parameters would have been around 0.048 mm/year. This value is intermediate between the erosion rates of basaltic terrains in the tropics (Hawaii^77^) and higher than mafic terrains in temperate climates (Czech Republic^78^), suggesting a less arid climate in the region during this time.

### Regional effects of the Mesopotamian Seaway closure

Based on our model results, the Maltese record points to a sharp decrease in the flux of water flowing from the Indian Ocean across the Mesopotamian Seaway by an order of magnitude from >20 Sv to ~2 Sv during the Early Burdigalian (ca. 20 Ma). Considering contributions from the Western Mediterranean volcanism would allow for a more significant reduction in contributions from the Indian Ocean source, as these would have made the Atlantic component more radiogenic. However, this would also require higher more radiogenic neodymium isotope inputs along the Mesopotamian Seaway to compensate for the comparatively less radiogenic input from the Western Mediterranean sources.

Given that global sea level was overall rising at that time^79^, we suggest that this change must have been tectonically driven and was coeval with the initiation of the Anatolian block exhumation^80^ movement along the Dead Sea Transform^81^, and initiation of continental crust collision between the Arabian and Eurasian plates^82^. The emplacement of evaporites along the Mesopotamian Seaway first occurred at ca. 22 Ma in the Qom Basin (Southern Iran) followed by a second phase during the earliest Burdigalian (ca. 20.4 Ma)^9,83^. The timing of these depositional events coincided with a drop in seawater ɛNd observed in Malta as well as in northern Italy, and both events are separated by a peak in the radiogenic ɛNd signatures observed in the late Aquitanian Maldives record (Fig. 2). These unradiogenic signatures may have resulted from short and transient periods of restricted exchange via the Mesopotamian Seaway.

The time of initiation of sapropel formation in the East Mediterranean Sea has been estimated to 16 and 13.5 Ma^84,85^, while upwelling in the western Arabian Sea started between about 15 and 13 Ma^86,87^. Such events would have been impossible in the presence of a well-connected marine seaway with significant exchange of both surface and intermediate waters. The seawater ɛNd values observed in the Maltese record until ca.14 Ma (Fig. 3) suggest that a shallow connecting seaway with significantly restricted water mass exchange still persisted until the late Langhian. These two marked steps in the ɛNd signatures (early Burdigalian and Langhian-Serravallian boundary) are not clearly observed in the northern Italian record, possibly due to weathering contributions from local volcanic rocks^21^ in conjunction with the coeval circulation patterns in the western Mediterranean. The northern Italian section may also have been influenced to some extent by contributions from the Paratethys which exhibit ɛNd values between −8 and −10 in the Alpine regions^88,89^, and are thus slightly more radiogenic than Atlantic sources. Further to the east, emplacement of significant evaporitic deposits in northern Iraq and Syria continued at least until 14 Ma^90,91^, also indicating that a shallow connection to the Indian Ocean was probably still present until this time. Our dataset shows that the Mediterranean ɛNd values from the Serravallian to the Messinian Salinity Crisis remained below −9, which is consistent with models that show the dominance of Atlantic waters without any exchange with the Indian Ocean, either with or without contribution for Western Mediterranean volcanism. This range of values may also have been partially modulated by contribution from the Paratethys, although the differences between the Northern Italian record and the Maltese record suggest that these effects were more significant in the northern part of the Mediterranean relative to its southern part.

The final establishment of a permanent ice sheet on Antarctica caused a significant drop in sea level and contributed to the decoupling of the Mediterranean and Paratethys seas^92^. In the eastern Mediterranean this was coeval to a slab steepening below the Anatolian block close to the Langhian-Serravalian boundary (Mi3b isotope excursion at 13.8 Ma^93,94^), and resulted in the final separation between the Indian Ocean and the Mediterranean Sea.

### Implications for ocean heat transfer and global climate

The closure of the Tethyan seaway resulted in the termination of global oceanic exchange of surface waters at low latitudes. This physical restriction resulted in a major redistribution of salt and heat which affected current and climate patterns in the North Atlantic and Indian Ocean. Although the exact causal relationships are not fully understood, it likely also affected the Southern Ocean and the ACC, as suggested by models^5^. The new age constraints on the closure presented here may allow an improved evaluation of the impact on the strength of the ACC. A more direct effect was likely the enhancement of the Mediterranean Outflow Water (MOW) and as a consequence a modification the AMOC^95,96^ and the Atlantic salinity gradient with possible climatic effects^97^–^100^.

In the Indian Ocean, however, the reduction of the inflow of warm and salty waters of the Tethyan Indian Saline Waters (TISW) from the Mediterranean Sea together with a possibly stronger circulation in the Southern Ocean paved the way to the initiation of upwelling in the Arabian Sea^86^, although it was not until 13 Ma that indications for hypoxia appear^87^. These changes were accompanied by a major reorganisation of currents in the Indian Ocean at that time^101^ and likely also contributed to the establishment of the modern South Asian Monsoon. Based on recent work, the SAM initiated in a weaker state at ca. 20 Ma and then intensified to reach its modern pattern and strength by 13 Ma^27,28^. This timing overlaps with the two pulses of reduction of connectivity to the Mediterranean and we speculate that the decrease in the heat leakage from the western Indian Ocean to the Mediterranean was one of the dominating mechanisms.

It is unclear how far the effect of reduced TISW advection reached, which may have been restricted to the Arabian sea and the eastern African Margin^102^. Due to that the TISW signal may have been diluted outside the Arabian Sea and the east of the African margin, which would explain why it is not clearly recorded at Site 707 on the Mascarene Plateau^101^. In the aftermath of the second phase of closure at ca. 13 Ma, a highly radiogenic intermediate water signal is observed across the lower latitudes of Indian ocean^48,101,103^. This signal is attributed to local strong intermediate water currents derived from the Pacific Ocean (“MIOjets”)^101^ labeled by exchange with the volcanic islands of Indonesia through which the currents were routed.

The onset of the permanent glaciation of Antarctica overlapped with the second phase^104^ and thus it is unclear if the Antarctic glaciation or the closure of the gateway played the more important role in the later reorganization of the SAM system. We speculate that the closure, in combination with the formation of the jet systems^101^, resulted in trapping of thermal energy in the northwestern Indian Ocean and increased sea surface temperatures, which was an important component of the intensification of the SAM through increasing the rate of energy transfer from the sea surface to the atmosphere^105,106^.

The connection between the Indian Ocean and the Mediterranean defined the oceanic circulation in both basins until its termination, first by tectonic forcing, and then as a consequence of the global sea level drop. The gateway closure may have had a positive feedback effect on global cooling and precipitation patterns by intensifying the ACC and the AMOC as well as through rearrangement of the heat and salt budget of the northern Indian Ocean, thereby directly affecting the SAM.

## Supplementary information

Supplement to Two-step closure of the Miocene Indian Ocean Gateway to the Mediterranean
