# Supplementary material for: Two-step closure of the Miocene Indian Ocean Gateway to the Mediterranean
Source: Sci Rep. 2019 Jun 20;9:8842. doi: 10.1038/s41598-019-45308-7 (PMC6586870; doi:10.1038/s41598-019-45308-7)
Supplement: Supplementary file 1 — Supplement to Two-step closure of the Miocene Indian Ocean Gateway to the Mediterranean [file 41598_2019_45308_MOESM1_ESM.pdf]

Supplement to Two-step closure of the Miocene Indian Ocean Gateway to the Mediterranean

Bialik, Or M.[1*]; Frank, Martin[2]; Betzler, Christian[3]; Zammit, Raymond[4]; Waldmann, Nicolas D.[1]

[1] Dr. Moses Strauss Department of Marine Geosciences, The Leon H. Charney School of Marine Sciences, University of Haifa, Carmel 31905, Israel.

[2] GEOMAR Helmholtz Centre for Ocean Research Kiel, Kiel, Germany.

[3] Institute of Geology, CEN, University of Hamburg, Bundesstrasse 55, Hamburg 20146, Germany.

[4] The School of Earth and Ocean Sciences, Cardiff University, Cardiff, Wales, UK

*Corresponding author (orbialik@campus.haifa.ac.il)

Analytical methods

For Nd isotope analyses of past seawater from ferromanganese coatings of the sediment particles, the bulk sediment samples consisting mainly of nannofossil and planktonic foraminifer oozes, and chalks were dried and homogenised in an agate mortar. To extract the authigenic, seawater-derived Nd isotope signature, approximately 2.5 g of powdered bulk sediment was treated following the procedure described in Gutjahr et al. (2007) omitting the carbonate removal step. The powdered samples were rinsed three times with de-ionized (MQ) water, after which 10 ml of MQ was added and 10 ml of a 0.05M hydroxylamine hydrochloride/15% acetic acid solution, buffered with NaOH to a pH of 4. Samples were placed on a shaker for 1 hour and centrifuged. The supernatant containing the seawater Nd isotope signature of the ferromanganese coatings was pipetted off and dried down. As preparatory steps for column chemistry, all samples were refluxed in concentrated HNO_3 at 80°C overnight, centrifuged, and 80% of the supernatant was dried down. Twice, 0.5 ml of 1 M HCl was added, and the sample was dried down, after which the samples were redissolved in 0.5 ml 1 M HCl. Samples were passed through cation-exchange columns with 0.8 ml AG50W-X12 resin (mesh size 200–400 μm), using standard procedures, to separate Sr and the Rare Earth Elements (REEs), as well as removing most of the Ba (Barrat et al., 1996). A second set of columns with 2 ml Ln-Spec resin (mesh size 50–100 μm) was used to separate Nd from the other REEs and remaining Ba (Le Fèvre and Pin, 2005).

Neodymium isotope ratios were measured on a Neptune Multiple Collector Inductively Coupled Plasma Mass Spectrometer (MC-ICPMS) at GEOMAR Kiel, Germany. Measured $^{143}Nd/^{144}Nd$ results were mass-

bias corrected to a $^{146}Nd/^{144}Nd$ ratio of 0.7219 and were normalised to the accepted $^{143}Nd/^{144}Nd$ value of 0.512115 for the JNdi-1 standard (Tanaka et al., 2000), which was measured after every third sample.

Nd isotope ratios are reported as ε_{Nd} values with respect to the Chondritic Uniform Reservoir (CHUR), which are calculated as $\varepsilon_{Nd} = [(^{143}Nd/^{144}Nd)_{sample} / (^{143}Nd/^{144}Nd)_{CHUR} - 1] * 10^4$ using a $(^{143}Nd/^{144}Nd)_{CHUR}$ value of 0.512638. No correction of the $^{143}Nd/^{144}Nd$ for ingrowth of ^{143}Nd from ^{147}Sm in the samples was carried out given that the difference is at maximum 0.25 εNd units for the oldest samples. The external reproducibility (2σ) of the measurements was between 0.14 and 0.25 ε_{Nd} units. The internal 2σ error was applied when larger than the external reproducibility. Procedural Nd blanks were ≤30 pg Nd and thus negligible.

Table S1: εNd values from Malta and Site U1468

Site	Age	Age Reference	εNd(t)	2σ
Fomm Ir Rih	23.40	Föllmi et al. 2008	-5.05	0.26
il-Blata	22.05	Föllmi et al. 2008	-4.30	0.14
il-Blata	21.12	Föllmi et al. 2008	-4.90	0.14
il-Blata	21.10	Baldassini and Di Stefano, 2015	-4.55	0.14
il-Blata	19.91	Föllmi et al. 2008	-4.23	0.15
il-Blata	19.39	Föllmi et al. 2008	-7.64	0.15
il-Blata	16.95	Baldassini and Di Stefano, 2015	-8.23	0.14
il-Blata	14.20	Föllmi et al. 2008	-8.75	0.15
Gnejna Bay	15.00	Föllmi et al. 2008	-8.78	0.17
Gnejna Bay	14.06	Föllmi et al. 2008	-9.72	0.14
Gnejna Bay	13.82	Abels et al., 2005	-8.73	0.14
Gnejna Bay	13.68	Abels et al., 2005	-10.82	0.14
U1468A	12.75	Betzler et al., 2016	-7.96	0.25
U1468A	13.30	Betzler et al., 2016	-8.16	0.25
U1468A	13.68	Betzler et al., 2016	-8.00	0.25
U1468A	14.06	Betzler et al., 2016	-8.78	0.25
U1468A	14.66	Betzler et al., 2016	-7.73	0.25
U1468A	14.84	Betzler et al., 2016	-8.79	0.25
U1468A	15.07	Betzler et al., 2016	-7.54	0.19
U1468A	15.56	Betzler et al., 2016	-7.21	0.79
U1468A	16.07	Betzler et al., 2016	-7.52	0.25
U1468A	16.36	Betzler et al., 2016	-7.89	0.25
U1468A	16.54	Betzler et al., 2016	-7.29	0.25
U1468A	17.28	Betzler et al., 2016	-8.05	0.25
U1468A	17.43	Betzler et al., 2016	-7.78	0.25
U1468A	17.86	Betzler et al., 2016	-8.00	0.25
U1468A	18.03	Betzler et al., 2016	-7.00	0.25
U1468A	18.36	Betzler et al., 2016	-6.80	0.25
U1468A	18.98	Betzler et al., 2016	-8.71	0.25
U1468A	19.21	Betzler et al., 2016	-7.53	0.25
U1468A	19.50	Betzler et al., 2016	-7.29	0.42
U1468A	20.10	Betzler et al., 2016	-7.87	0.25
U1468A	20.55	Betzler et al., 2016	-5.22	0.25
U1468A	20.78	Betzler et al., 2016	-5.20	0.41
U1468A	21.47	Betzler et al., 2016	-7.13	0.25
U1468A	24.25	Betzler et al., 2016	-10.52	0.25
U1468A	24.44	Betzler et al., 2016	-9.39	0.81
U1468A	24.63	Betzler et al., 2016	-7.55	0.55
U1468A	25.04	Betzler et al., 2016	-10.64	0.31
U1468A	25.28	Betzler et al., 2016	-7.33	0.26

Box model

In order to better constrain our observations, a simple box model of the Mediterranean was established. The water balance of the basin was defined by equation 1:

$$1)\quad \frac{dW}{dt} = F_{Atlantic} + F_{Indian} + F_{Agean} + F_{Rivers} - F_{Evaporation} - F_{outflow}$$

Where F is the volume flux of water in Sv (10^6 m^3 sec^{-1}), Indian Ocean and Atlantic Ocean influx were set at initial conditions following the results of modelling work (de la Vara et al., 2013; de la Vara and Meijer, 2016) at 22.64 Sv and 4.78 Sv, respectively. Evolving conditions of the Atlantic inlet were defined by a fit of the relationships between the Indian and Atlantic inlet in the different modelling experiments (Fig. S1). Due to uncertainty regarding the exchange with the Paratethys and the proto Aegean Sea, two modern values of pre- and post-East Mediterranean Transient (EMT) of 0.35 Sv to 1.2 Sv (Roether and Klein, 1998; Roether et al., 2007), respectively, were used in two different runs of the model. Riverine influx was estimated at 0.025 Sv (Simon et al., 2017). Operating under the assumption of a constant volume for the Mediterranean (3.75×10^{14} m^3) set to modern water flux values, mass was balanced to be setting the outflux equal to total influx minus evaporation (set to the modern value of 0.08 Sv following Shaltout and Omstedt, 2015) as defined by equation 2:

$$2)\quad F_{outflow} = \sum F_{in}^{i} - F_{evporation}$$

The neodymium concentration of the box was defined by equation 3 and εNd by equation 4:

$$3)\quad \frac{d[Nd]}{dt} = F_{Atlantic}[Nd]_{Atlantic} + F_{Indian}[Nd]_{Indian} + F_{Agean}[Nd]_{Agean} + F_{Rivers}[Nd]_{Rivers} - F_{outflow}[Nd]_{outflow}$$

$$4)\quad \frac{d\varepsilon Nd}{dt} = F_{Atlantic}[Nd]_{Atlantic}\varepsilon Nd_{Atlantic} + F_{Indian}[Nd]_{Indian}\varepsilon Nd_{Indian} + F_{Agean}[Nd]_{Agean}\varepsilon Nd_{Agean} + F_{Rivers}f_{Nile}[Nd]_{Rivers}\varepsilon Nd_{Nile} + F_{Rivers}(1 - f_{Nile})[Nd]_{Rivers}\varepsilon Nd_{Rhone} - F_{outflow}[Nd]_{outflow}\varepsilon Nd_{Med}$$

Where f_{Nile} represents the fraction of the total freshwater supply supplied by the Nile River (based on pre-1900 values; Said, 1993). Neodymium concentration and εNd values for each of the water sources are detailed in Table S2. Given that no concentration data are available for the Nile, it was assumed they are similar to that of the Rhone. Based on ferromanganese crust data (O'Nions et al., 1998) and the results of this study for the western Indian Ocean, present-day values of εNd for the Indian Ocean appear to be reasonable for the Miocene. Results of this version of the run are shown in figure S2. Further experiments carried out with the model using different values for the possible contribution sources (based on other sources noted in the text as well as observed values for the Maldives from this data set) have failed to reproduce the observed range of the Early Miocene from the Maltese record (Fig. S3).

To account for a possible volcanic contribution along the gateway itself a modification of the Indian Ocean flux component was introduced resulting in the following equations:

5) $$\frac{d[Nd]}{dt} = F_{Atlantic}[Nd]_{Atlantic} + F_{Indian}([Nd]_{Indian} + [Nd]_{volA}/10^3 F_{Indian}) + F_{Agean}[Nd]_{Agean} + F_{Rivers}[Nd]_{Rivers} - F_{outflow}[Nd]_{outflow}$$

6) $$\frac{d\varepsilon Nd}{dt} = F_{Atlantic}[Nd]_{Atlantic}\varepsilon Nd_{Atlantic} + F_{Indian}\left([Nd]_{Indian}\varepsilon Nd_{Indian} + \frac{[Nd]_{volA}\varepsilon Nd_{volA}}{10^3 F_{Indian}}\right) + F_{Agean}[Nd]_{Agean}\varepsilon Nd_{Agean} + F_{Rivers} f_{Nile}[Nd]_{Rivers}\varepsilon Nd_{Nile} + F_{Rivers}(1 - f_{Nile})[Nd]_{Rivers}\varepsilon Nd_{Rhone} - F_{outflow}[Nd]_{outflow}\varepsilon Nd_{Med}$$

Where Nd_{volA} is the total contribution of the neodymium introduced into to the seaway mixed with Indian Ocean waters along the northern Arabian Plate, and εNd_{volA} represents the corresponding εNd, which was set at +5, the median value of all the potential sources (Lease and Abdel-Rahman, 2008; Azizi and Moinevaziri, 2009; Trifonov et al., 2011; Ma et al., 2013). The results of this iteration are shown in figure 3 of the main text. In order to contribute the needed amount of radiogenic Nd as observed in the Early Miocene of Malta, some 0.2 mol/sec were needed to be supplied along the conduit. Assuming an area of 2 x 10^5 km^2, a mean Nd content of 31.5 ppm and basalt density of 3 g / cm^3 the erosion rate required would be 0.048 mm/year.

The model was run for 250 years from the initial modern value of the Eastern Mediterranean to steady state. The steady state values were used as initial conditions for subsequent runs during which F_{Indian} was diminished stepwise from the initial value of 22 Sv to 0 Sv. Each iteration of the diminishing flux runs was run for 250 years to allow for a steady state to be established.

To account for a possible contribution from a western Mediterranean source, we also allowed for contribution from a source along the Atlantic source:

7) $$\frac{d[Nd]}{dt} = F_{Atlantic}([Nd]_{Atlantic} + [Nd]_{volS}/10^{3}F_{Atlantic}) + F_{Indian}([Nd]_{Indian} + [Nd]_{volA}/10^{3}F_{Indian}) + F_{Agean}[Nd]_{Agean} + F_{Rivers}[Nd]_{Rivers} - F_{outflow}[Nd]_{outflow}$$

8) $$\frac{d\varepsilon Nd}{dt} = F_{Atlantic}\left([Nd]_{Atlantic}\varepsilon Nd_{Atlantic} + \frac{[Nd]_{volS}\varepsilon Nd_{volS}}{10^{3}F_{Atlantic}}\right) + F_{Indian}\left([Nd]_{Indian}\varepsilon Nd_{Indian} + \frac{[Nd]_{volA}\varepsilon Nd_{volA}}{10^{3}F_{Indian}}\right) + F_{Agean}[Nd]_{Agean}\varepsilon Nd_{Agean} + F_{Rivers}f_{Nile}[Nd]_{Rivers}\varepsilon Nd_{Nile} + F_{Rivers}(1 - f_{Nile})[Nd]_{Rivers}\varepsilon Nd_{Rhone} - F_{outflow}[Nd]_{outflow}\varepsilon Nd_{Med}$$

Where Nd_{volS} is the total contribution of the neodymium introduced to water coming from the Atlantic and εNd_{volS} represents the corresponding εNd, which was set at -4, the median value based on sources in Sardinia (Downes et al., 2001). This western Mediterranean source was scaled to half the Arabian source in the experiments. These values represent a maximum value used to estimate the highest impact. For illustration, figure S4 shows the output of a model experiment using the maximum erosion input rate. While there is some dampening of the trend by this source, its contribution is not highly significant nor changes the outcomes in any significant manner even at this high relative contribution.

Table S2: Modern neodymium composition of the Mediterranean and source end members

	εNd	[Nd] (pmol/kg)	Reference
Nile discharge	-1.25±0.25	?	(Scrivner et al., 2004)
Rhone discharge	-10.8±0.6	85.9±57.1	(Ayache et al., 2016) and references therein
Aegean Sea	-1.96±2.14	28.47±18.20	
East Mediterranean (surface + intermediate)	-6.57±1.42	30.94±4.35	(Tachikawa et al., 2004; Vance et al., 2004)
Indian Ocean	-7.99±1.07	16.13±8.92	(Bertram and Elderfield, 1993; Pomiès et al., 2002)
Atlantic inflow (surface)	-10.36±0.78	23.94±5.99	(Spivack and Wasserburg, 1988; Tachikawa et al., 2004)

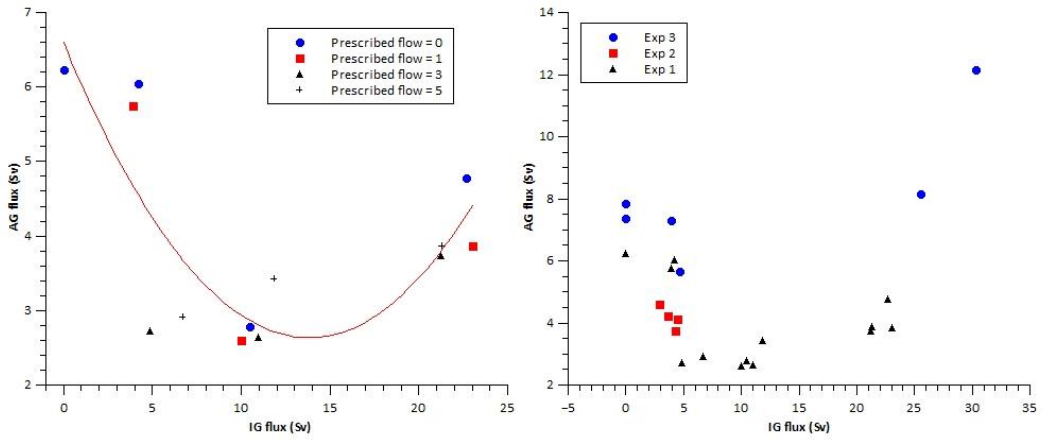

Figure S1: Relation between influx from the Indian (IG) and Atlantic (AG) Oceans into the Mediterranean based on published model simulations (de la Vara et al., 2013; de la Vara, 2015; de la Vara and Meijer, 2016)

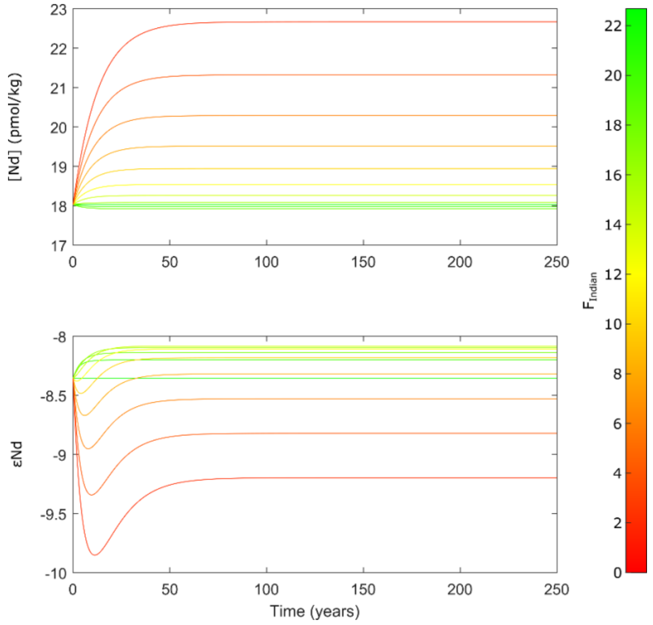

Figure S2: model results for Nd concentration and εNd in the Mediterranean using Indian ocean fluxes (F $_{Indian}$) and composition of water as described in Table S1.

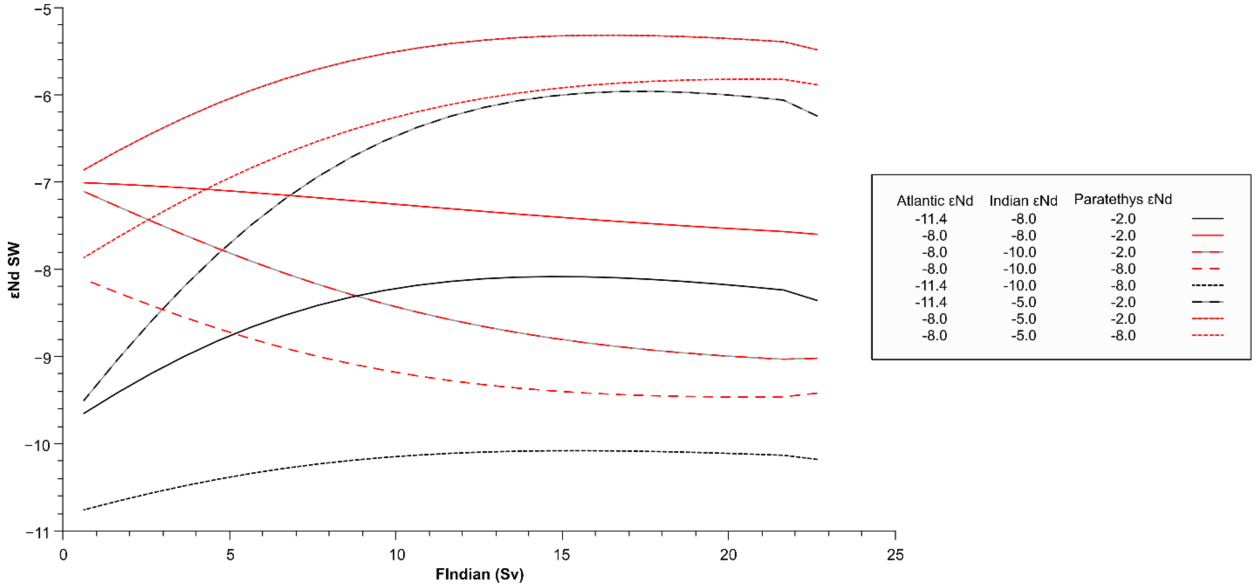

Figure S3: Partial outputs of different runs of the model where the εNd of the inputs was changed.

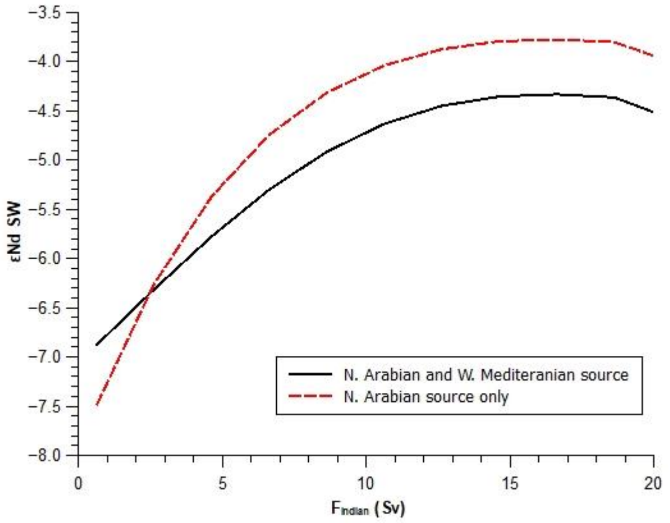

Figure S5: Model output results comparing changes in the εNd of Mediterranean seawater along a diminishing contribution from the Indian Ocean with a northern Arabian Plate contribution and a combination of the western Mediterranean and northern Arabian Plate contribution.

Additional figures

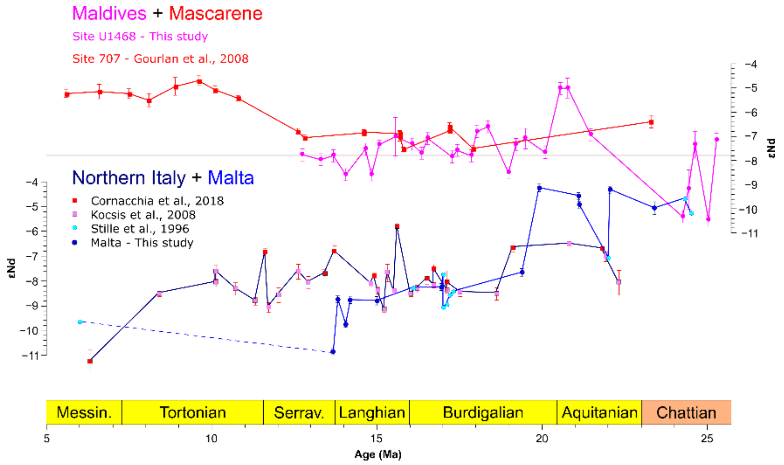

Figure S6: Compilation of all εNd from the Indian Ocean and the Mediterranean discussed in this manuscript.

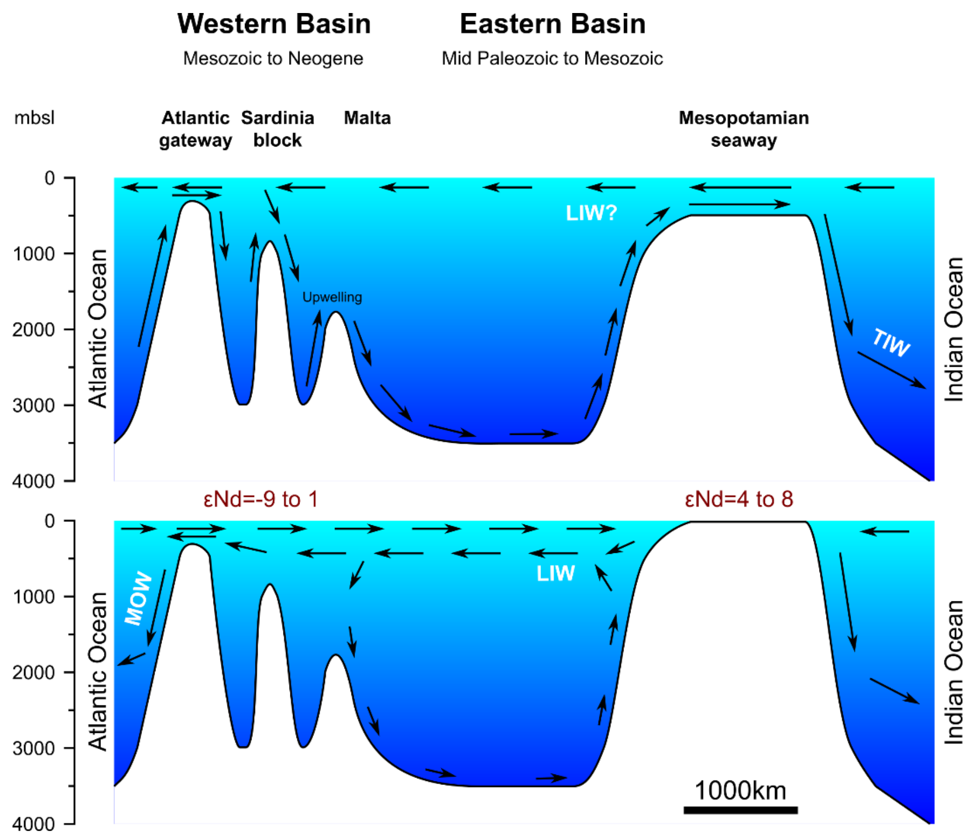

Figure S7: Schematic illustration of the main circulation patterns in the Mediterranean and in either gateway before (upper) and after (lower) decoupling from the Indian Ocean. Directions are based on Hamon et al. (2013); de la Vara et al. (2013); de la Vara (2015) and de la Vara and Meijer (2016). εNd values listed refer to values of exposed volcanic rocks in the marked locations; see text.

References:

Ayache M., Dutay J. C., Arsouze T., Révillon S., Beuvier J. and Jeandel C. (2016) High-resolution neodymium characterization along the Mediterranean margins and modelling of Nd distribution in the Mediterranean basins. *Biogeosciences* **13**, 5259–5276.

Azizi H. and Moinevaziri H. (2009) Review of the tectonic setting of Cretaceous to Quaternary volcanism in northwestern Iran. *J. Geodyn.* **47**, 167–179.

Barrat J. A., Keller F., Amossé J., Taylor R. N., Nesbitt R. W. and Hirata T. (1996) Determination of rare earth elements in sixteen silicate reference samples by ICP-MS after tm addition and ion exchange separation. *Geostand. Geoanalytical Res.* **20**, 133–139.

Bertram C. J. and Elderfield H. (1993) The geochemical balance of the rare earth elements and neodymium isotopes in the oceans. *Geochim. Cosmochim. Acta* **57**, 1957–1986.

Downes H., Thirlwall M. . and Trayhorn S. . (2001) Miocene subduction-related magmatism in southern Sardinia: Sr–Nd- and oxygen isotopic evidence for mantle source enrichment. *J. Volcanol. Geotherm. Res.* **106**, 1–22. Available at: http://linkinghub.elsevier.com/retrieve/pii/S0377027300002699.

Le Fèvre B. and Pin C. (2005) A straightforward separation scheme for concomitant Lu–Hf and Sm–Nd isotope ratio and isotope dilution analysis. *Anal. Chim. Acta* **543**, 209–221. Available at: http://linkinghub.elsevier.com/retrieve/pii/S0003267005006860.

Gutjahr M., Frank M., Stirling C. H., Klemm V., van de Flierdt T. and Halliday A. N. (2007) Reliable extraction of a deepwater trace metal isotope signal from Fe–Mn oxyhydroxide coatings of marine sediments. *Chem. Geol.* **242**, 351–370.

Hamon N., Sepulchre P., Lefebvre V. and Ramstein G. (2013) The role of eastern tethys seaway closure in the middle miocene climatic transition (ca. 14 Ma). *Clim. Past* **9**, 2687–2702.

de la Vara A. (2015) *Model Analysis of the Role of Marine Gateways in the Palaeoceanography of the Miocene Mediterranean and Paratethys*., Utrecht studies in Earth Sciencies, No. 98, Utrecht.

de la Vara A. and Meijer P. (2016) Response of Mediterranean circulation to Miocene shoaling and closure of the Indian Gateway: A model study. *Palaeogeogr. Palaeoclimatol. Palaeoecol.* **442**, 96–109.

de la Vara A., Meijer P. T. and Wortel M. J. R. (2013) Model study of the circulation of the Miocene Mediterranean Sea and Paratethys: closure of the Indian Gateway. *Clim. Past Discuss.* **9**, 4385–4424.

Lease N. A. and Abdel-Rahman A. F. M. (2008) The Euphrates volcanic field, northeastern Syria: Petrogenesis of Cenozoic basanites and alkali basalts. *Geol. Mag.* **145**, 685–701.

Ma G. S.-K., Malpas J., Suzuki K., Lo C.-H., Wang K.-L., Iizuka Y. and Xenophontos C. (2013) Evolution and origin of the Miocene intraplate basalts on the Aleppo Plateau, NW Syria. *Chem. Geol.* **335**, 149–171.

O’Nions R. ., Frank M., von Blanckenburg F. and Ling H.-F. (1998) Secular variation of Nd and Pb isotopes in ferromanganese crusts from the Atlantic, Indian and Pacific Oceans. *Earth Planet. Sci. Lett.* **155**, 15–28.

Pomiès C., Davies G. R. and Conan S. M. H. (2002) Neodymium in modern foraminifera from the Indian Ocean: Implications for the use of foraminiferal Nd isotope compositions in paleo-oceanography. *Earth Planet. Sci. Lett.* **203**, 1031–1045.

Roether W. and Klein B. (1998) The great eastern Mediterranean deep-water transient. *Rapp. Comm. Int. Mer Méditérranée* **35**, 12–16.

Roether W., Klein B., Manca B. B., Theocharis A. and Kioroglou S. (2007) Transient Eastern Mediterranean deep waters in response to the massive dense-water output of the Aegean Sea in the 1990s. *Prog. Oceanogr.* **74**, 540–571.

Said R. (1993) *The River Nile. Geology, Hydrology and Utilization*., Pergamon, Oxford.

Scrivner A. E., Vance D. and Rohling E. J. (2004) New neodymium isotope data quantify Nile involvement in Mediterranean anoxic episodes. *Geology* **32**, 565–568.

Shaltout M. and Omstedt A. (2015) Modelling the water and heat balances of the Mediterranean Sea using a two-basin model and available meteorological, hydrological, and ocean data. *Oceanologia* **57**, 116–131.

Simon D., Marzocchi A., Flecker R., Lunt D. J., Hilgen F. J. and Meijer P. T. (2017) Quantifying the Mediterranean freshwater budget throughout the late Miocene: New implications for sapropel formation and the Messinian Salinity Crisis. *Earth Planet. Sci. Lett.* **472**, 25–37.

Spivack A. J. and Wasserburg G. J. (1988) Neodymium isotopic composition of the Mediterranean outflow and the eastern North Atlantic. *Geochim. Cosmochim. Acta* **52**, 2767–2773.

Tachikawa K., Roy-Barman M., Michard A., Thouron D., Yeghicheyan D. and Jeandel C. (2004) Neodymium isotopes in the Mediterranean Sea: Comparison between seawater and sediment signals. *Geochim. Cosmochim. Acta* **68**, 3095–3106.

Tanaka T., Togashi S., Kamioka H., Amakawa H., Kagami H., Hamamoto T., Yuhara M., Orihashi Y., Yoneda S., Shimizu H., Kunimaru T., Takahashi K., Yanagi T., Nakano T., Fujimaki H., Shinjo R., Asahara Y., Tanimizu M. and Dragusanu C. (2000) JNdi-1: a neodymium isotopic reference in consistency with LaJolla neodymium. *Chem. Geol.* **168**, 279–281.

Trifonov V. G., Dodonov A. E., Sharkov E. V., Golovin D. I., Chernyshev I. V., Lebedev V. A., Ivanova T. P., Bachmanov D. M., Rukieh M., Ammar O., Minini H., Al Kafri A. M. and Ali O. (2011) New data on the Late Cenozoic basaltic volcanism in Syria, applied to its origin. *J. Volcanol. Geotherm. Res.* **199**, 177–192.

Vance D., Scrivner A. E., Beney P., Staubwasser M., Henderson G. M. and Slowey N. C. (2004) The use of foraminifera as a record of the past neodymium isotope composition of seawater. *Paleoceanography* **19**, 1–17.
